# Influence of Persian Gum and Almond Gum on the Physicochemical Properties of Wheat Starch

**DOI:** 10.3390/gels9060460

**Published:** 2023-06-03

**Authors:** Sara Hedayati, Elham Ansarifar, Mohammad Tarahi, Zahra Tahsiri, Vahid Baeghbali, Mehrdad Niakousari

**Affiliations:** 1 Nutrition Research Center, School of Nutrition and Food Sciences, Shiraz University of Medical Sciences, Shiraz 7193635899, Iran; 2 Social Determinants of Health Research Center, Department of Public Health, School of Health, Birjand University of Medical Sciences, Birjand 9717853076, Iran; ansarifar.elham@bums.ac.ir; 3 Department of Food Science and Technology, School of Agriculture, Shiraz University, Shiraz 7144165186, Iran; tarahimohammad@yahoo.com (M.T.); zahratahsiri@yahoo.com (Z.T.); 4 Food and Markets Department, Natural Resources Institute, University of Greenwich, Medway, Kent ME4 4TB, UK; v.baeghbali@greenwich.ac.uk

**Keywords:** wheat starch, almond gum, persian gum, physicochemical properties

## Abstract

In this study, the influence of different levels (0.1, 0.2, and 0.3% *w*/*w*) of Persian gum or almond gum were incorporated into wheat starch, and their influences on water absorption, freeze–thaw stability, microstructure, pasting, and textural properties were investigated. The SEM micrographs revealed that the addition of hydrocolloids to starch leads to the formation of denser gels with smaller pores. The water absorption of starch pastes was improved in the presence of gums, and samples containing 0.3% almond gum had the highest water absorption. The rapid visco analyzer (RVA) data showed that the incorporation of gums significantly affected the pasting properties by increasing the pasting time, pasting temperature, peak viscosity, final viscosity, and setback and decreasing breakdown. In all the pasting parameters, the changes caused by almond gum were more obvious. Based on TPA measurements, hydrocolloids were able to improve the textural properties of starch gels, such as firmness and gumminess but decreased the cohesiveness, and springiness was not affected by the incorporation of gums. Moreover, the freeze–thaw stability of starch was enhanced by the inclusion of gums, and almond gum exhibited better performance.

## 1. Introduction

Starch is the main constituent of wheat flour and is considered a major source of energy in the human diet. Wheat starch is the third most used starch in the world after corn and cassava starches which is stored in water-insoluble granules with semi-crystalline structures. It is a mixture of two polymers of D-glucose, a linear *α*-(1→4) linked molecule called amylose, and a highly branched structure of *α*-(1→4) linked glucan with *α*-(1→6) branching links, termed amylopectin. Amylose accounts for approximately 20–30%, while amylopectin accounts for approximately 70–80% of starch granules. The properties of wheat starch determine the quality of flour-based products such as bread, noodles, pasta, cakes, cookies, etc. Additionally, functional properties such as water absorption, emulsion stabilization, pasting, and gelation have changed wheat starch into a unique hydrocolloid with several applications in food products. However, native wheat starch has inherent properties such as poor resistance to thermal and shear stresses, tendency to retrogradation, instability in cold–storage, sensitivity to freezing and acids, and relatively low water absorption capacity and paste viscosity. These properties deteriorate the quality of food products and prevent the extensive application of native starch in the food industry. In order to take advantage of starch and enhance its applications in processed food products, its functional properties can be modified by different methods such as chemical, enzymatic and physical treatments [1]. Among these modification methods, chemically modified starches have gained wider applications in the food industry mainly due to the existence of a large number of hydroxyl groups on starch chains which provide active sites for functional groups such as carboxyl, acetyl, hydroxypropyl, amine, amide, etc. These functional groups have the potential to improve the functional and nutritional properties of modified starches and provide a wide range of physicochemical benefits. Nevertheless, many consumers are concerned about the presence of chemical reagents in chemically modified starches. On the other hand, enzymatic modifications may lead to the formation of a variety of hydrolysates, undesirable taste and texture, and thus, cannot be applied extensively. Therefore, physical treatments are preferable and have great potential for starch modification when compared to chemical and enzymatic modification methods. Some physical treatments, such as the incorporation of natural ingredients into native starches, might be a promising option for improvement in starch functionality. Mixing starch with hydrocolloids is considered an inexpensive and safe modification technique. Hydrocolloids are hydrophilic biopolymers with a neutral taste, which are commonly used in different food products. A combination of starch and non-starch polysaccharides may be used in food matrices to enhance the quality of food products by improving their freeze–thaw stability, water binding capacity, hindrance of ice crystallization, emulsion stability, sensory attributes as well as their nutritional properties [2,3,4,5,6].

The effects of hydrocolloid incorporation on the physicochemical and nutritional properties of starch have been investigated extensively. The effects of gum Arabic (GA) on tapioca starch (TS) were investigated by Singh et al. [7]. They found that the elastic modulus (G’) and the viscous modulus (G”) were increased by GA, while peak viscosity and setback were decreased. They also detected an increase in consistency coefficient and apparent viscosity with the increase in GA concentrations. Lutfi et al. [8] studied the effects of xanthan, guar, gum acacia, and carboxymethyl cellulose (CMC) on pasting and functional features of water chestnut starch (WCS). They found that the inclusion of these hydrocolloids increased the solubility and syneresis but decreased the swelling power. The RVA data also displayed significant changes in pasting properties in the presence of gums. The pasting temperature was decreased with CMC, and the setback was enhanced by xanthan gum but reduced with gum acacia, and xanthan gum increased breakdown. The majority of published reports are focused mainly on the commonly used food hydrocolloids. However, in recent years some types of gums, such as tragacanth, are becoming more and more expensive while others, such as gum Arabic, are not sustainable due to the crisis in Senegal, Sudan, and Somalia, which are the major producing countries [9]. Therefore, there have been increasing demands for natural, safe, renewable, available, and biodegradable novel hydrocolloids in the food industry. In accordance with this trend, scientists have investigated the potential applications and capabilities of new sources of natural gums as substitutes for the existing common gums. Gum exudates are novel and inexpensive sources of hydrocolloids which are composed of long-chain polysaccharides comprising aldopentoses, aldohexoses, and uronic acids linked by glycoside bonds [10]. Persian gum is the exudate of *Amygdalus scoparia Spach* shrubs which are mostly grown in central regions of Iran [11]. Persian gum has diverse applications in traditional medicines for the treatment of chronic coughs, bladder stones, toothaches, and headaches [12]. Properties such as high-water absorption, low cost, and availability make it a potential alternative for the commonly used gums in the food industry. Persian gum can have potential applications in food products as thickening, adhering, emulsifying, suspending, and stabilizing agents [13]. Almonds (*Amygdalus communis* L.) are a member of Rosaceae family that grows in the Mediterranean region, southwest Asia, and the Middle East [14]. The trunk and branches of almond trees produce clear gum exudates in white, yellow, and red colors. The exudation of gum from almond trees is due to gummosis disease or mechanical injury [15]. This hydrocolloid has great water absorption, apparent viscosity, and emulsifying properties; thus, it can be used in different food products [16]. However, almond gum has received less attention which resulted in the high wastage of this hydrocolloid [17]. Since wheat starch is one of the most widely used sources of starch and the main constituent of wheat flour, introducing new hydrocolloids to overcome the shortcomings of native wheat starch is necessary. Therefore, the objective of this study was to examine the influence of Persian gum and almond gum on the functional properties of native wheat starch.

## 2. Results and Discussion

### 2.1. Water Absorption

The influence of gums on the water absorption of wheat starch is given in Table 1. Both gum exudates increased the water absorption of starch, and it was enhanced by the concentration of gums. The same results were observed by Yousefi et al. [18] when sage seed gum was added to wheat starch. However, Chaisawang et al. [19] reported an opposite trend for starch-xanthan gum mixtures and stated that xanthan gum inhibits the swelling of starch by preventing amylose leaching. The structure of carbohydrates has a principal role in the improvement of water absorption of starch. Starch pastes containing almond gum had higher water absorption compared to pastes with Persian gum. The molecular weight, the degree of polar hydroxyl groups in polysaccharides, and the extent of hydrodynamic interactions affect the water absorption of polysaccharides [20]. Almond gum had higher molecular weight and hydroxyl groups compared to Persian gum. Therefore, water molecules interact with the gum molecules more feasibly by hydrogen bonding, and it has a higher water absorption capacity compared to Persian gum [16,21].

### 2.2. Pasting Properties

The pasting parameters of starch and gum mixtures are shown in Table 2. Compared with native starch, the pasting parameters of samples containing gums were markedly higher. The pasting temperature of the control sample was 65.30 °C and increased significantly with the addition of Persian gum and almond gum. Almond gum had a greater impact on pasting temperature (69.92 °C) compared to Persian gun (67.18 °C). Pasting temperature shows the beginning of gelatinization and is the temperature at which viscosity increases. Von Borries-Medrano et al. [22] reported the same results after the addition of tara gum, guar gum, and locust bean gum to tapioca starch. Additionally, Lan and Lai [23] observed a similar trend for water caltrop starch after the incorporation of konjac glucomannan, guar gum, and xanthan gum. They stated that the pasting temperature and peak time was increased by increasing the concentration of gums and attributed this behavior to the competition between starch and gums for the available water. The increase in pasting temperature is related to a decrease in free water. Persian gum and almond gum absorbed and immobilized water and acted as competitors for available water. Therefore, water was deficient around starch granules, and the pasting temperature was increased. Almond gum had higher water absorption (Table 1); therefore, the pasting temperature of samples with almond gum was higher than Persian gum. Furthermore, the addition of gums increased the pasting time, implying that starch gelatinization was retarded. Apparently, gum exudates improve the heat stability of starch and hinder molecular disruption. The presence of gums significantly (*p* ˂ 0.05) increased the peak viscosity, which indicates that the water absorption of starch was increased in the presence of gums. The highest peak viscosity was observed in samples with 0.3% almond gum, which is in line with the results of the water absorption test (Table 2). The improvement of peak viscosity by the addition of polysaccharides has been reported by Ma et al. [24] for corn starch in the presence of konjac glucomannan and Ma et al. [25] for corn starch with pectin. However, Rong et al. [26] reported that the peak viscosity of potato starch was decreased in the presence of *Mesona chinensis* Benth gum and hypothesized that gum covers the surface of potato starch granules and leads to the reduction of water-absorption and expansion capacity of starch granules. The breakdown is defined as the fall in viscosity after peak viscosity and is a result of the disintegration of starch granules during heating and shearing in RVA experiment. The incorporation of gum exudates into starch decreased the breakdown values, which means that these hydrocolloids increased the heat stability of starch granules. Setback shows the increase of viscosity after cooling the starch paste and is an indication of reaggregation of starch granules and short-term retrogradation, while final viscosity is the viscosity of the starch paste at the end of the RVA experiment. Final viscosity and setback express the tendency of starch to form a gel. These parameters were increased in the presence of hydrocolloids. Gum exudates form strong interactions with the amylose chains in the aqueous phase and increase the final viscosity and setback values.

### 2.3. Textural Properties

The textural parameters of starch gels containing different levels of gums are presented in Table 3. When the starch paste is cooled down, a gel network is formed. Gel hardness is generally associated with water absorption of starch. As can be seen, the gel hardness was increased with the concentration of gums, and samples containing almond gum showed higher hardness than those with Persian gum. The linear structure of amylose molecules reassociates and forms a three-dimensional network. The hardness of wheat starch was 539.74 g and reached 805.18 g and 705.07 g in starch gels with 0.3% of almond gum and Persian gum, respectively. Similar results were reported by Shams–Abadi and Razavi [27] for wheat starch-cress seed gum mixtures. The increase in hardness of starch gels in combination with hydrocolloids can be attributed to higher water absorption of gels in the presence of gums. Cohesiveness shows the resistance of starch gel structure after deformation. The hydrocolloid-containing starch gel represented lower cohesiveness values than the control sample, which indicate lower resistance to textural damage. Amylose content is the key factor that contributes to the formation of starch gel and its textural properties. During the gelatinization of starch, starch granules swell, and amylose chains leach out of them. When the starch paste is cooled, interactions are formed and develop into a cohesive gel [28]. The lower cohesiveness of starch gels in the presence of gums may be attributed to lower amylose leaching of starch granules into the aqueous phase. Hydrocolloids surround starch granules and prevent amylose leaching and the formation of a cohesive network [29,30]. Moreover, non-covalence cross junctions, which participate in the formation of cohesive gels, cannot be formed in low concentrations of gums because many of the intermolecular zones cannot contribute to the formation of junction zones. Therefore, low levels of hydrocolloids cannot significantly improve the macromolecular networks [31]. Gumminess is a result of hardness × cohesiveness and is defined as the amount of energy required to break a semisolid food in order to make it ready for swallowing and is a characteristic of semisolid foods [32]. Among hydrocolloid-containing gels, those with almond gum showed higher gumminess than those with Persian gum, which indicates that these gels need more energy to become ready for swallowing. The gumminess of wheat starch was 410.11, while it was 538.67 and 478.74 for gels containing 0.3% almond gum and Persian gum, respectively. Almond gum had higher water absorption than Persian gum (Table 1) and reduced the available water, which in turn led to moisture binding by gums and the formation of gels. Springiness shows the elasticity of starch gels, and this parameter was not significantly impacted by the addition of gums.

### 2.4. Freeze–thaw Stability

**Figure 1:**
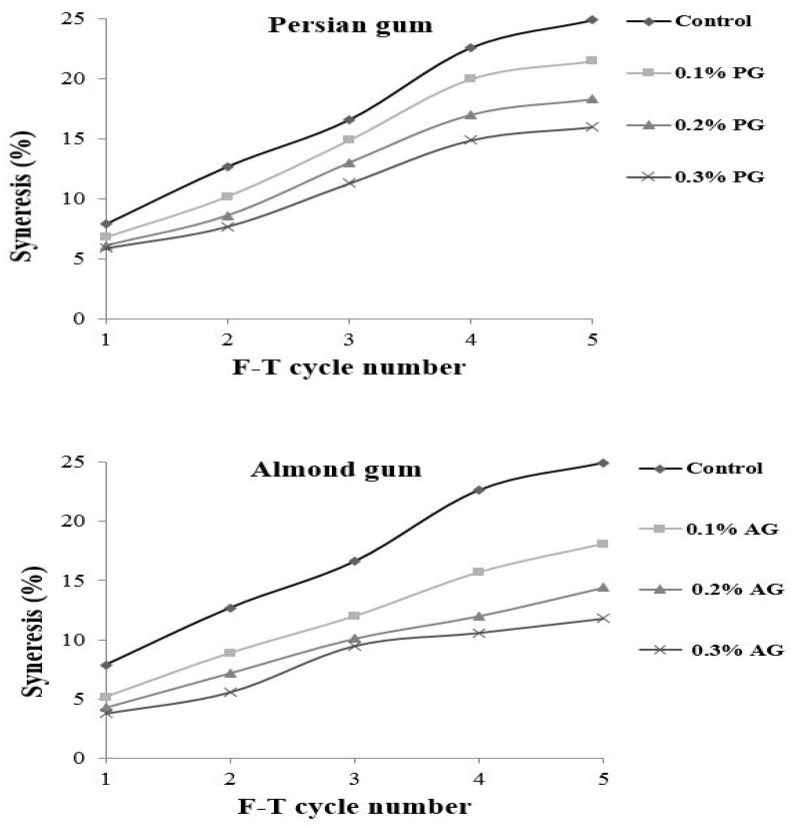
Syneresis (%) of wheat starch gels containing different concentrations of Persian gum and almond gum.

The freeze–thaw stability shows the capacity of starch to resist temperature shocks. During freeze–thaw cycles, water is released from a starch gel network due to the rearrangement of the leached starch molecules. It is an undesirable phenomenon and can be used as an indication of starch retrogradation. The freeze–thaw stability of wheat starch containing different levels of gums is presented in Figure 1. The results revealed that wheat starch without gum was not stable during Freeze–thaw cycles and showed the highest amount of separated water. The incorporation of gums decreased the water release, and samples with almond gum showed lower water separation. The amount of separated water was increased by repeating Freeze–thaw cycles for all of the samples. When the starch matrix is freeze–thawed several times starch network structure is destroyed and forms starch-rich regions; therefore, large ice crystals are developed and result in high water release upon thawing [33,34]. Increasing the gum concentration decreased the water separation. Polysaccharides have a hydrophilic nature and decrease the amount of water released. The effects of almond gum on improving the freeze–thaw stability of starch were more significant than those presented by Persian gum. The higher water absorption of almond gum compared to Persian gum is due to an improvement in the degree of bound water and suggests that almond gum has better cryo-preservative effects.

### 2.5. Microstructure of Gels

**Figure 2:**
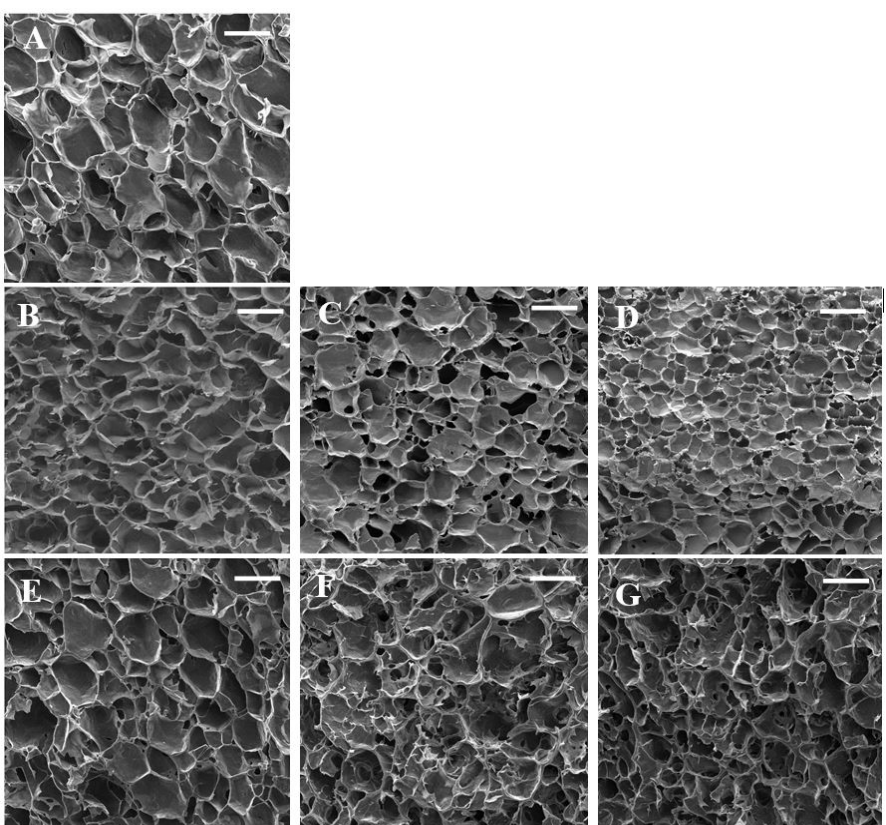
SEM micrographs of wheat starch gels containing different concentrations of gums. (**A**) (wheat starch), (**B**) (wheat starch + 0.1% almond gum), (**C**) (wheat starch + 0.2% almond gum), (**D**) (wheat starch + 0.3% almond gum), (**E**) (wheat starch + 0.1% Persian gum), (**F**) (wheat starch + 0.2% Persian gum), (**G**) (wheat starch + 0.3% Persian gum). (Scale bar = 100 µm).

The SEM micrographs are presented in Figure 2. The control sample showed a honeycomb-like structure. Sun et al. [35] reported a similar structure for wheat starch gels. This three-dimensional network led to the elasticity of starch gel and its ability to hold water. The swelling of starch granules during heating led to amylose leaching, and these leached amylose chains are responsible for the formation of starch gel during cooling [26]. The microstructure of starch gels was significantly affected by the addition of gums. Control starch gel had larger pores with thinner cell walls which are in line with textural parameters. Nevertheless, the addition of gum exudates formed denser gels with thicker cell walls. The sample containing 0.3% almond gum had the smallest pores due to its high water absorption capacity. These findings are in agreement with the results reported by Shams–Abadi and Razavi [27] for wheat starch-cress seed gum mixtures. They stated that increasing the concentration of gum decreased the size of pores, and the gel structure became denser. When the concentration of gums increases, the gum–amylose matrix fills the spaces between starch granules and develops a dense and interconnected honeycomb structure. However, Rong et al. [36] stated that the addition of guar, xanthan, and *Mesona chinensis* Benth gums increased the pore size of pea starch gels. According to their observations, pea-starch–gum mixtures had a compact structure, and it was difficult to separate water from gels; subsequently, large and irregular holes were developed in the starch gel matrix.

## 3. Conclusions

The results of this study revealed that the incorporation of gum exudates, such as Persian gum and almond gum can improve the microstructure, freeze–thaw stability, water absorption, pasting, and textural properties of wheat starch. These changes were more obvious by increasing the concentration of gums, and in all of the measured parameters, almond gum was more effective than Persian gum. Therefore, almonds can be a better potential candidate to be used in starch-based food products in order to improve their functional properties.

## 4. Materials and Methods

### 4.1. Materials

Wheat starch was obtained from Fars–Glucosin Co. (Marvdasht, Iran) that contained 7% moisture, 0.44% fat, 0.38% protein, and 0.13% ash (determined by the approved methods of the AACC [37]. The total amylose content of wheat starch was 26.20%, as measured by the method described by Hoover and Ratnayake [38]. Persian gum was collected from the shrubs of wild almonds. It contained 8.87% moisture, 2.34% ash, 0.21% fat, 0.19% protein, 88.39% total sugar, and 9.73% uronic acid. The average molecular weight of Persian gum was 4.15 × 10^6^ Da. The almond gum was collected from the trunk and branches of sweet almond trees in Shiraz, Iran. It contained 7.35% moisture, 0.94% ash, 0.30% fat, 0.38% protein, 87. 12% total sugars, and 2.84% uronic acid, and the average molecular weight was 11.19 × 10^6^ Da.

### 4.2. Water Absorption

Water absorption was determined by the method described by Rahimi et al. [29]. In brief, suspensions (3% *w*/*v*) were prepared by incorporating starch with distilled water or gum solutions. The suspensions were prepared with a magnetic stirrer (LABINCO L-81, Breda, Breda, The Netherlands). The samples were heated in a water bath at 95 °C for 30 min under constant agitation (200 rpm) and then cooled to ambient temperature and centrifuged (Sigma 3–30 KS, Osterod am Harz, Germany) at 700 g for 20 min. The separated water was removed, and the sediment was weighed to determine the water absorption with the following equation:

$$Water\;absorption\;(g/g)=\frac{Weight\;of\;starch\;paste\;residue}{Initial\;weight\;of\;starch\;powder}$$

### 4.3. Freeze–Thaw Stability

The freeze–thaw stability of starch pastes was measured according to the method described by Tarahi et al. [39]. The wheat starch powder was weighed in screw-cap centrifuge tubes, and appropriate amounts of distilled water or gum solutions were added to them to form (5%) slurries. Starch–gum suspensions were heated at 95 °C for 30 min in a water bath under constant stirring. Subsequently, the prepared samples were frozen in a freezer at −18 °C for 24 h and thawed at 30 °C for 4 h. Freeze–thaw cycles were repeated five times, and after each cycle, the pastes were centrifuged at 8000× *g* for 15 min, and the released water from each paste was weighed, and syneresis (%) was quantified by the following equation:

$$Syneresis\;(\%)=\frac{Weight\;of\;released\;water}{Initial\;weight\;of\;starch\;paste}\times 100$$

### 4.4. Pasting Properties

The pasting properties of wheat starch-gum composites were determined by using a rapid visco analyzer (RVA) (StarchMaster 2, Perten Instruments, Sydney, New South Wales, Australia). 3.5 g of starch powder was added to 25 g of water or gums solutions (0.1, 0.2, and 0.3%) in an RVA canister and inserted into the instrument. The slurry was heated at 50 °C for 1 min, then heated to 95 °C and kept at 95 °C for 2.7 min, and then cooled to 50 °C at the rate of 5 °C/min. The paddle rotation speed was 960 rpm for the first 10 s and then reached 160 rpm. Pasting parameters, including pasting temperature, pasting time, peak viscosity, breakdown, setback, and final viscosity, were determined from the RVA curves.

### 4.5. Texture Profile Analysis

The pastes obtained from the RVA were transferred into Plexiglas molds (20 mm inner diameter, 10 mm height), covered to avoid moisture loss, and stored at 4 °C for 24 h before textural measurements. Gels were removed from holders, and texture profile analysis (TPA) was performed using a texture analyzer (TA-XT2, Stable Micro Systems, Surrey, UK) with a cylindrical probe (35 mm diameter). The gel mixtures were compressed to 25% of their original height at a crosshead speed of 0.25 mm/s. Textural parameters such as hardness (maximum force required for a 25% compression in the first compression cycle), cohesiveness (the ratio between the area under the second peak and the area under the first peak), springiness (the height that the starch gels recovered during the time between the end of the first compression cycle and the start of the second compression cycle), and gumminess (hardness × cohesiveness) were obtained from time-force curves using the Texture Exponent Lite software (version 5.1.1.0) [29].

### 4.6. Scanning Electron Microscopy

The microstructure of the starch-gum composite gels was studied using a scanning electron microscope (TESCAN-Vega 3, Brno, Czech Republic). The samples were freeze-dried (Dena Vacuum, Tehran, Iran). Cross sections of samples were prepared by a sharp knife and coated with gold in a sputter coater (Quorum Technologies, Q150R-ES, East Sussex, UK). The micrographs were taken under vacuum conditions with an accelerating voltage of 15 kV and 300× magnification.

### 4.7. Statistical Analysis

The statistical analysis of the experimental data was performed by one-way analysis of variance (ANOVA) at a confidence level of 0.05. The means were compared using Duncan’s multiple range test at a significance level of 0.05 with SPSS statistical software version 16.

## Figures and Tables

**Table 1:** Water absorption (g/g) of wheat starch containing different concentrations of Persian gum and almond gum.

Treatment	Gum Concentration (%)	Water Absorption (g/g)
Control	0	8.04 ± 0.037 ^f^
Almond gum	0.1	8.55 ± 0.093 ^d^
	0.2	9.38 ± 0.024 ^c^
	0.3	10.15 ± 0.045 ^a^
Persian gum	0.1	8.22 ± 0.081 ^e^
	0.2	8.65 ± 0.052 ^d^
	0.3	9.64 ± 0.13 ^b^

Values are expressed as the average of three replications ± standard deviations. Means with different superscript letters are significantly different from each other (*p* ˂ 0.05).

**Table 2:** Pasting properties of wheat starch containing different concentrations of Persian gum and almond gum.

Treatment	Gum Concentration (%)	Pasting Temp. (°C)	Pasting Time (min)	Peak Viscosity (cP)	Breakdown (cP)	Setback (cP)	Final Viscosity (cP)
Control	0	65.29 ± 0.14 ^e^	1.030 ± 0.028 ^f^	2837 ± 36.77 ^f^	1614 ± 17.68 ^a^	1845 ± 39.59 ^d^	3068 ± 58.68 ^e^
Almond gum	0.1	65.90 ± 0.07 ^d^	1.133 ± 0.034 ^de^	3329 ± 11.69 ^d^	1541 ± 4.61 ^b^	1818 ± 3.92 ^d^	3606 ± 5.02 ^d^
	0.2	67.18 ± 0.11 ^b^	1.499 ± 0.042 ^c^	3651 ± 38.28 ^b^	1399 ± 3.05 ^c^	1928 ± 50.98 ^c^	4180 ± 18.10 ^b^
	0.3	69.92 ± 0.09 ^a^	2.068 ± 0.047 ^a^	3871 ± 39.92 ^a^	1382 ± 39.11 ^c^	2140 ± 8.93 ^a^	4629 ± 12.92 ^a^
Persian gum	0.1	65.40 ± 0.20 ^e^	1.051 ± 0.026 ^ef^	3049 ± 34.11 ^e^	1614 ± 12.98 ^a^	1884 ± 17.12 ^cd^	3319 ± 2.09 ^e^
	0.2	66.47 ± 0.13 ^c^	1.240 ± 0.050 ^d^	3310 ± 32.03 ^d^	1581 ± 39.07 ^ab^	1948 ± 27.71 ^c^	3677 ± 19.17 ^c^
	0.3	67.18 ± 0.25 ^b^	1.619 ± 0.055 ^b^	3477± 41.51 ^c^	1409 ± 11.96 ^c^	2072 ± 15.11 ^b^	4140 ± 39.75 ^b^

Values are expressed as the average of three replications ± standard deviations. Means in the same column with different superscript letters are significantly different (*p* ˂ 0.05).

**Table 3:** Textural properties of wheat starch gels containing different concentrations of Persian gum and almond gum.

Treatment	Gum Concentration (%)	Hardness (g)	Cohesiveness	Springiness	Gumminess (g)
Control	0	539.74 ± 15.87 ^d^	0.760 ± 0.018 ^a^	0.983 ± 0.003 ^a^	410.11 ± 18.38 ^d^
Almond gum	0.1	640.18 ± 19.14 ^c^	0.746 ± 0.012 ^a^	0.983 ± 0.003 ^a^	477.44 ± 12.62 ^b^
	0.2	709.02 ± 25.52 ^b^	0.710 ± 0.011 ^b^	0.989 ± 0.007 ^a^	503.39 ± 16.93 ^b^
	0.3	805.18 ± 35.31^a^	0.669 ± 0.006 ^c^	0.984 ± 0.004 ^a^	538.67 ± 28.09 ^a^
Persian gum	0.1	529.83 ± 16.29 ^d^	0.749 ± 0.009 ^a^	0.986 ± 0.005 ^a^	396.84 ± 17.33 ^d^
	0.2	629.31 ± 7.05 ^c^	0.714 ± 0.015 ^b^	0.990 ± 0.006 ^a^	449.33 ± 3.33 ^c^
	0.3	705.07 ± 17.06 ^b^	0.679 ± 0.009 ^c^	0.983 ± 0.007 ^a^	478.74 ± 8.07 ^b^

Values are expressed as the average of three replications ± standard deviations. Means in the same column with different superscript letters are significantly different (*p* ˂ 0.05).

## Data Availability

The data presented in this study are available upon request from the corresponding author.
